# Arthroscopic cystectomy and valve excision of popliteal cysts complemented with management of intra-articular pathologies: a low recurrence rate and good functional outcomes in a series of ninety seven cases

**DOI:** 10.1007/s00264-023-05745-6

**Published:** 2023-03-13

**Authors:** Konrad Malinowski, Marcin Mostowy, Michał Ebisz, Przemyslaw A. Pękala, Nicholas I. Kennedy, Robert F. LaPrade

**Affiliations:** 1 Department of Anatomy, International Evidence-Based Anatomy Working Group, Jagiellonian University Medical College, Kraków, Poland; 2 Artromedical Orthopaedic Clinic, Antracytowa 1, 97-400 Belchatow, Poland; 3 Orthopedic and Trauma Department, Veteran’s Memorial Teaching Hospital in Lodz, Medical University of Lodz, Zeromskiego 113, 90-549 Lodz, Poland; 4 Faculty of Medicine and Health Sciences, Andrzej Frycz Modrzewski Kraków University, Kraków, Poland; 5 Lesser Poland Orthopedic and Rehabilitation Hospital, Kraków, Poland; 6 Twin Cities Orthopedics, 4010 W 65th St Edina, 55435 Minnesota, USA

**Keywords:** Popliteal cyst, Baker’s cyst, Arthroscopy

## Abstract

**Purpose:**

Arthroscopy in popliteal cyst surgery enables addressing all components of its pathomechanism: the cyst wall, valvular mechanism, and concomitant intra-articular pathologies. Techniques differ as to the management of the cyst wall and the valvular mechanism. This study aimed to assess the recurrence rate and functional outcomes of a cyst wall and valve excising arthroscopic technique with concurrent intra-articular pathology management. The secondary purpose was to assess cyst and valve morphology and concomitant intra-articular findings.

**Methods:**

Between 2006 and 2012, 118 patients with symptomatic popliteal cysts refractory to at least three months of guided physiotherapy were operated on by a single surgeon using a cyst wall and valve excising arthroscopic technique with intra-articular pathology management. Patients were evaluated preoperatively and at a mean follow-up of 39 months (range 12–71) by ultrasound, Rauschning and Lindgren, Lysholm, and VAS of perceived satisfaction scales.

**Results:**

Ninety-seven out of 118 cases were available for follow-up. Recurrence was observed on ultrasound in 12/97 cases (12.4%); however, it was symptomatic only in 2/97 cases (2.1%). Mean scores improved: Rauschning and Lindgren from 2.2 to 0.4, Lysholm from 54 to 86, and VAS of perceived satisfaction from 5.0 to 9.0. No persistent complications occurred. Arthroscopy revealed simple cyst morphology in 72/97 (74.2%) and presence of a valvular mechanism in all cases. The most prevalent intra-articular pathologies were medial meniscus (48.5%) and chondral lesions (33.0%). There were significantly more recurrences in grade III–IV chondral lesions (*p* = 0.03).

**Conclusions:**

Arthroscopic popliteal cyst treatment had a low recurrence rate and good functional outcomes. Severe chondral lesions increase the risk of cyst recurrence.

## Introduction

The presence of a popliteal cyst (Baker’s cyst) in most cases is associated with concomitant intra-articular pathologies, which result in a persistent and extensive intra-articular effusion [1, 2]. This factor causes the progressive accumulation of the fluid within the gastrocnemius–semimembranosus bursa, whereas the valvular mechanism present at its entrance prevents the evacuation of the liquid back into the knee joint cavity. Taken together, this mechanism leads to progressive cyst formation and enlargement, subsequent symptoms arising from the popliteal region of the knee including pain and limitations of knee range of motion [3–5].

Traditionally, treatment of this condition consisted either of nonoperative treatment or open surgery through a posterior approach [6, 7]. Usually, the first line of popliteal cyst therapy consists of nonoperative management, with ultrasound-guided corticosteroid injections being a widespread method [5]. Although this treatment modality could be effective in the case of simple cysts, complex cysts are at risk of relapsing after conservative therapy [8]. Moreover, nonoperative treatment does not address the concomitant intra-articular lesions that cause the excessive synovial fluid production [1, 5]. As shown by a recent systematic review, both nonoperative treatment and open surgery resulted in a relatively high rate of cyst recurrence [5].

Currently, the treatment of popliteal cysts not only addresses the wall of the cyst but also targets the valvular mechanism and concomitant intra-articular pathologies, thus encompassing all components of its pathomechanism [3, 4]. Those principles are easily utilized in an arthroscopic setting due to direct visualization and treatment of intra-articular lesions. However, arthroscopic techniques of popliteal cyst surgery differ with regard to the management of the valvular mechanism (valve excision or closure surgery) and the cyst’s wall (excision or preservation) [3, 4].

This study aimed to assess the recurrence rate and functional outcomes of a cyst wall and valve excising arthroscopic technique with concurrent management of intra-articular pathologies. The secondary purpose was to assess cyst and valve morphology and concomitant intra-articular findings.

## Methods

### Study design and patients

Ethical approval was obtained from District medical chamber (approval number K.B.-27/2021). The study was designed in compliance with the Helsinki Declaration.

This was a retrospective cohort study of consecutive patients presenting to the single centre suffering from symptomatic popliteal cysts. Between 2006 and 2012, 118 consecutive patients (age, 18 to 57 years old) were included in the study and all data were collected prospectively in the database. The diagnosis of a popliteal cyst was based on the physical examination, ultrasonography, and magnetic resonance imaging (MRI) and confirmed during arthroscopy. Prior to surgery, all patients underwent at least three months of intensive guided physiotherapy with no improvement. The mean preoperative Rauschning and Lindgren scale of the popliteal cyst symptoms were 2.2 point out of possible 3 points (Table 1, the lower the better) [9], Lysholm score was 54 points out of possible 100 points (the higher the better) [10], and visual analog score (VAS) of perceived satisfaction was 5.0 points out of possible 10 points (the higher the better).

**Table 1:** Rauschning and Lindgren scale of the popliteal cyst symptoms

Grade	Description
0	No pain in the popliteal fossa and swelling, normal range motion
1	Swelling and pain after hard exercise with minimal reduction of range motion
2	Swelling and pain after soft exercise with reduction of range motion < 20°
3	Swelling and pain at rest with reduction of range motion > 20°

### Surgical technique

**Fig. 1:**
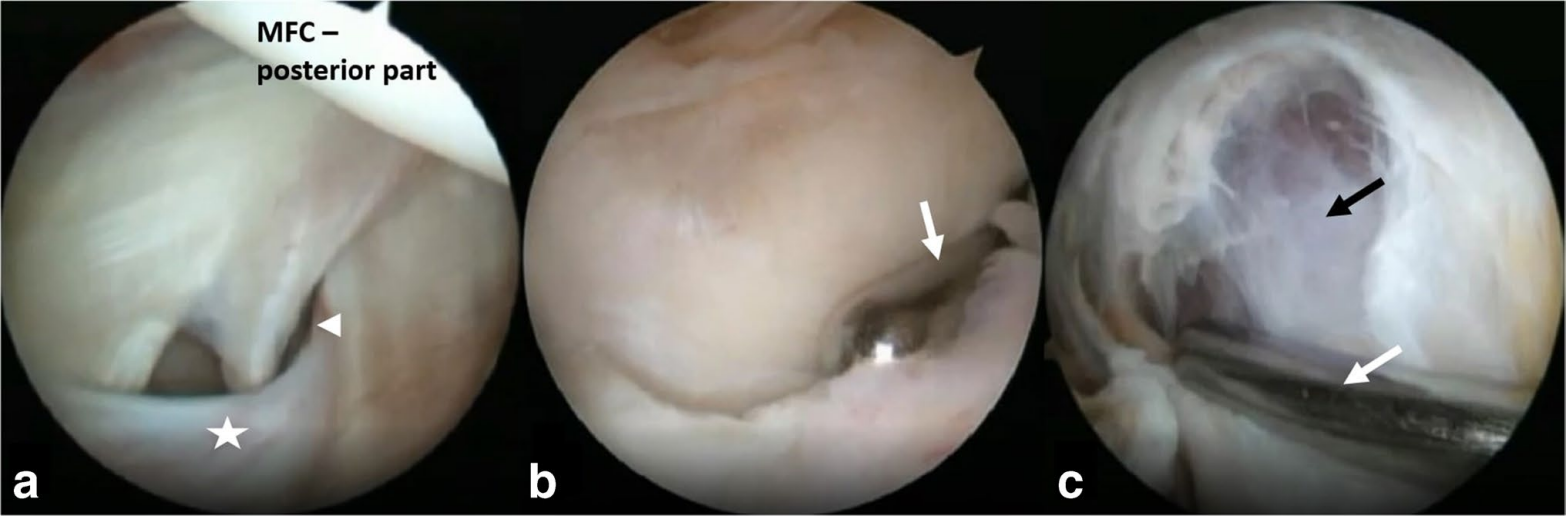
The excision of a popliteal cyst: (**a**) right knee—identification of the cyst’s entrance by lowering a posteromedial synovial fold (asterisks) with a needle (white arrowhead); (**b**) right knee—resection of the fold acting as valve with a shaver (white arrow); and (**c**) right knee—excision of the cyst’s wall with a shaver (white arrow); black arrow—fibers of semimembranosus muscle. MFC: medial femoral condyle

The procedure was performed by a single surgeon (*initials blinded for review*) according to a predefined, published technique (Fig. 1) [11, 12]. First, a diagnostic arthroscopy was performed and encountered intra-articular pathologies were treated. Then, the morphology of the cyst and the valvular mechanism connecting the cyst with the joint were evaluated and the “cyst procedure” was performed as the last part of the surgery. The transverse fold of the posteromedial joint capsule, usually acting as a valve at the entrance to the popliteal cyst, was identified using a spinal needle pierced through the posteromedial skin, and a posteromedial arthroscopic portal was made. A shaver and radiofrequency probe, introduced through the posteromedial portal, were subsequently used to excise the fold and to enlarge the communication between the cyst and the joint cavity. Then, the arthroscope was pushed into the cyst. The shaver was introduced through the posteromedial portal and guided subcutaneously to the medial wall of the cyst, which was subsequently excised. Finally, the shaver was pushed into the cyst and other walls of the cyst were excised [11, 12].

### Assessment of outcomes

Outcomes were assessed at a mean follow-up of 39 months (range 12–71 months). Ultrasonography of the popliteal fossa was used to evaluate for a cyst recurrence, which was defined as a presence of any amount of fluid within the gastrocnemius–semimembranosus bursa. Functional outcomes assessed included the Rauschning and Lindgren scale of the popliteal cyst symptoms (Table 1) [9], Lysholm scale [10], and the VAS of perceived patient satisfaction. Assessment of cyst and valve morphology and concomitant intra-articular findings was performed intraoperatively. Chondral lesions were assessed using the Outerbridge Classification System [13]. Associations of the recurrence rate with intra-articular pathologies presence, cyst morphology, and previous open procedure were analyzed statistically using the Fisher exact test. Data was collected in the years 2006–2012 using the predefined outcome sheets (Table 2).

**Table 2:** The morphology of the valvular mechanism of a popliteal cyst

Valvular mechanism	Quantity (%)
Present in typical location consisting of the following: ➢ Isolated transverse fold of the posteromedial capsule ➢ Massive synovial hypertrophy and a transverse fold	95/97(97.9%) 92/95 (96.8%) 3/95 (3.2%)
Coat-like shape of the posterior capsule	2/97 (2.1%)

## Results

Of the whole group, 21/118 patients (17.8%) were lost to follow-up. The remaining 97 patients were assessed at a mean follow-up of 39 months (range 12–71 months).

### Recurrence rate

**Fig. 2:**
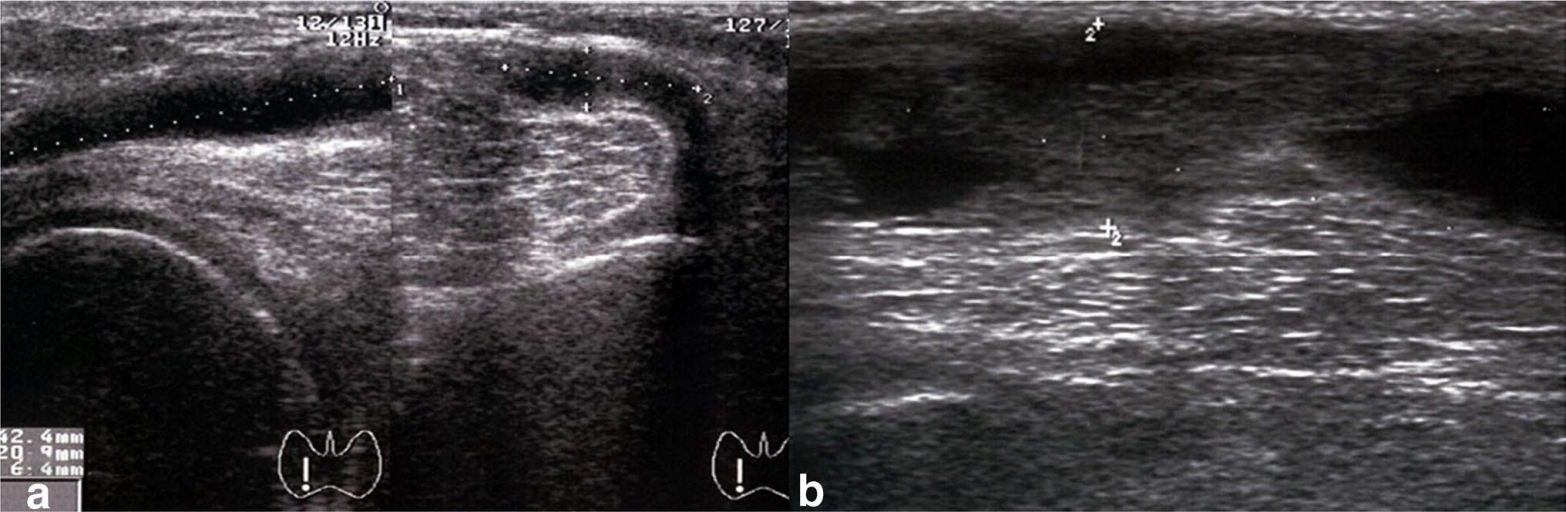
The recurrence of a popliteal cyst detected by means of the ultrasonography: (**a**) asymptomatic, slit-like cyst and (**b**) refilling and overgrowth of the cyst

The cyst was absent in 85 (87.6%) of 97 cases as assessed by means of ultrasonography. Therefore, an ultrasonographic recurrence rate was 12/97 (12.4%) (Fig. 2). For the remaining 12 patients with recurrence, in 10 cases, the cysts were asymptomatic and undetectable on physical examination. Therefore, the intervention was functionally successful in 95/97 (97.9%) of all cases. There were significantly more cyst recurrences in cases with III–IV Outerbridge grade chondral lesions than I–II grade (*p* = 0.03, Table 3). No significant correlation was detected between the recurrence of the cyst and any other intra-articular pathology, the multichamber morphology of the cyst, and the excision of the recurrent cysts as a revision after an open surgery.

**Table 3:** The coexisting intra-articular lesions and their association with recurrences

		Patients with recurrence											
Lesion	Quantity (% of whole group of 97 patients)	1	2	3	4	5	6	7	8	9	10	11	12
Medial meniscus tear	47/97 (48.5%)		✓	✓		✓			✓		✓		
Chondral lesions: Grade I–II Grade III–IV	32/97 (33.0%) 14/32 18/32												
					✓						✓		
		✓	✓	✓		✓	✓	✓	✓	✓		✓	✓
Instability	27/97 (27.8%)		✓						✓				
Synovitis and synovial hypertrophy	13/97 (13.4%)				✓								
Plica syndrome	6/97 (6.2%)										✓		
Lateral meniscus tear	5/97 (5.2%)		✓										
Rheumatoid diseases	3/97 (3.1%)				✓								
Chondromatosis	1/97 (1.0%)												
Osteochondritis dissecans	1/97 (1.0%)												

### Functional outcomes

The improvement in all functional outcome scores was noted. The mean Raushning–Lindgren scale of the popliteal cyst symptoms score decreased from a mean 2.2 points preoperatively to 0.4 at the final follow-up. The mean Lysholm score increased from 54 to 86 at the follow-up, and the mean VAS perceived satisfaction score increased from 5.0 to 9.0 at final follow-up. Postoperative popliteal pain resolved within a mean time of 28 h (defined as discontinuation of analgesic therapy) and a return of full knee range of motion occurred within 2.5 days on average.

### Cyst-related and intra-articular findings

**Fig. 3:**
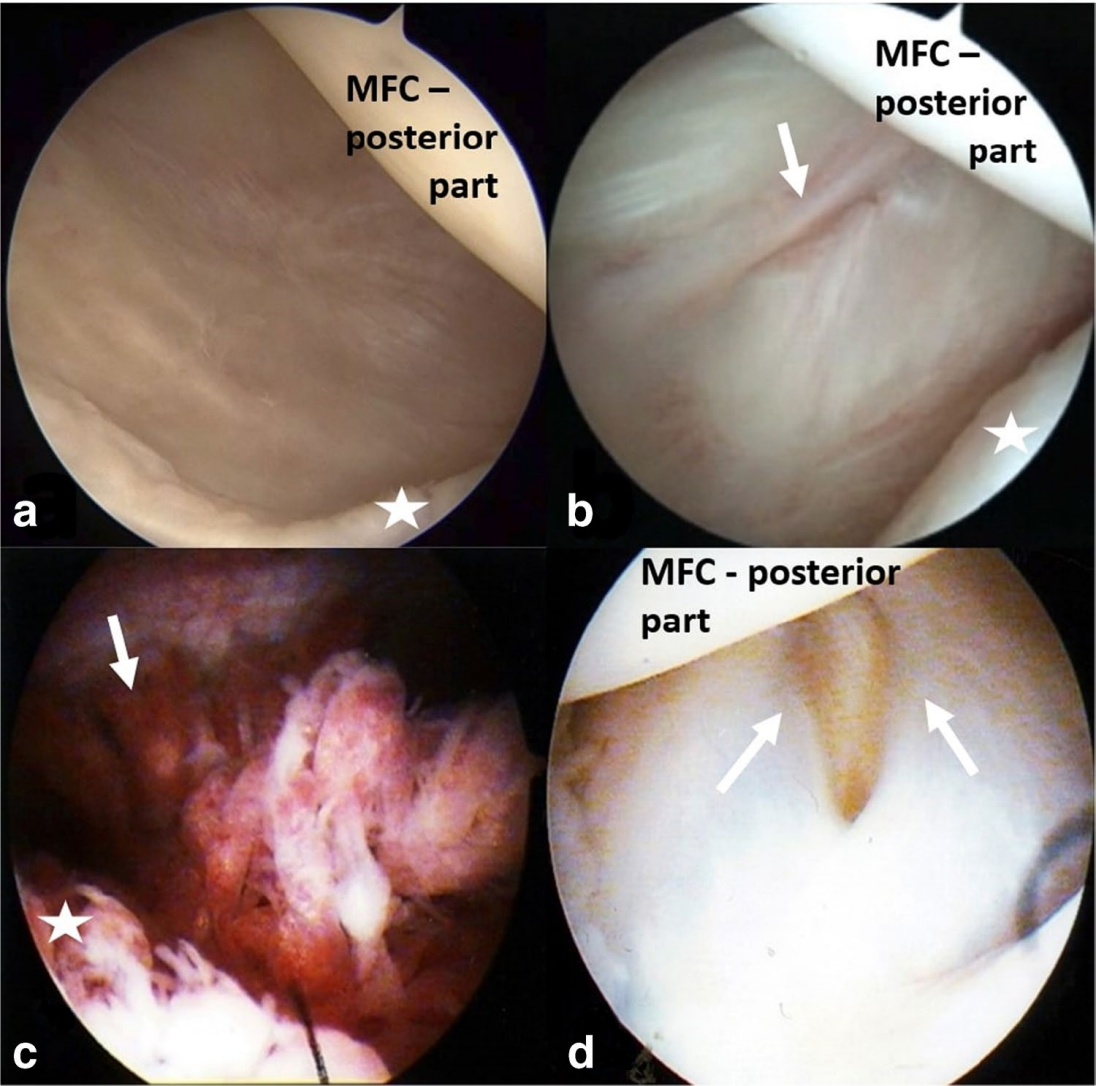
Different types of posteromedial capsule anatomy and popliteal cyst valvular mechanisms: (**a**) right knee—normal posteromedial capsule—no connection, asterisks—posterior horn of medial meniscus; (**b**) right knee—transverse synovial fold (white arrow) of the posteromedial capsule, asterisks—posterior horn of medial meniscus; (**c**) left knee—massive synovial hypertrophy (white arrow), asterisks—posterior horn of medial meniscus; and (**d**) left knee—coat-like shape of posteromedial capsule (white arrows)

Simple morphology of the cyst was present in 72/97 (74.2%) cases. Complex cysts, multichamber cysts, or cysts divided by a septum were present in 25/97 (25.8%) patients. Eight of 97 (8.2%) cases were recurrences after an open cyst excision. Arthroscopy revealed the communication between the cyst and joint cavity with valvular mechanism in all patients. The valvular mechanism was in most cases created by the posteromedial capsule transverse fold (97.9% of cases). The morphology of the valvular mechanism is presented in Table 2 and Fig. 3. The most prevalent intra-articular pathologies were medial meniscus tears (48.5%) and chondral lesions (33.0%). The distribution of concomitant intra-articular lesions and their association with recurrences is presented in Table 3.

### Safety

The postoperative complications included transient calf swelling in 8/97 (8.2%) of cases, popliteal haematoma in 5/97 (5.2%) of cases, transient sensory deficit near posteromedial portal in 2/97 (2.1%) cases, and superficial soft tissue infection in 1/97 (1.0%) case. All complications resolved within weeks after surgery.

## Discussion

The most important finding of this study was that an arthroscopic popliteal cyst excision technique led to a low recurrence rate and good functional outcomes. However, severe chondral lesions were associated with increased cyst recurrence risk.

Comparison of the recurrence rates from this study with the literature is difficult due to reporting discrepancies with regard to definition of the cyst recurrence and the modality used to assess it. Gu et al. reported a recurrence rate of 0% as assessed on MRI at 12–36 months of follow-up [14]. Ahn et al. reported that the cyst persisted on the MRI after arthroscopic surgical treatment in 45% of all cases at 12–72 months of follow-up [15]. Chen et al. described a one case (4.8% of the whole cohort) of a recurrence of a relatively large cyst (3 cm) detected on ultrasonography three months after surgery [16]. In a study by Lie et al., no recurrence was reported at a mean follow-up of 13 months (range 8–32), although patients were only assessed clinically [17]. A systematic review by Han et al. assessing outcomes in arthroscopic management of popliteal cysts found a recurrence rate of 0% when cystectomy was performed and a recurrence rate of 8% in those treated without concurrent cystectomy [18]. In the current study, the recurrence rate at a mean follow-up of 39 months (range 12–71) was 12.4% when assessed by the means of ultrasonography and 2.1% when assessed clinically. The possible reason for relatively high ultrasonographic recurrence rate in this study could be the restrictive ultrasonographic assessment criteria for cyst recurrence (any fluid in popliteal space), irrespectively of symptoms exhibited. Another possible reason could be inclusion of patients with advanced knee degenerative changes. What our study adds to existing literature is a significant association between the recurrence of the cyst and grade III–IV chondral lesions, probably due to the persistent excessive synovial fluid production [1, 5]. This finding could help physicians to further assess patients preoperatively for ideal surgical candidates.

In the current study, the popliteal functional outcomes after cyst excision were analyzed by the means of Rauschning and Lindgren score, Lysholm score, and VAS perceived satisfaction score. These scores were chosen in order to assess specifically popliteal symptoms by the means of Rauschning and Lindgren score as well as general symptoms of the knee by the means of the Lysholm score and the subjective satisfaction of the patients by the means of VAS perceived satisfaction score. What is more, in the current study, the assessed group covered many different reasons of popliteal cyst formation from sports lesions to advanced osteoarthritis and synovial diseases. Therefore, the authors chose not to use a score focused on a specific pathology. Gu et al., Ahn et al., Chen et al., and Lie et al. used the Rauschning and Lindgren score for assessment of functional outcomes. Despite differences in reporting, good functional improvement was achieved in those five arthroscopic studies [14–17]. Han et al. in their systematic review similarly assessed success via the Rauschning and Lindgren score findings comparable success to this work, with 185/186 patients undergoing arthroscopic treatment with cystectomy experiencing clinical success (RL grade 0 or 1) and 115/125 patients without cystectomy experiencing success [18]. As to the Lysholm score, Yang et al. reported the increase of the mean Lysholm score from preoperative 47.3 to 85.8 at the mean follow-up of 13.8 months [7], and Jiang et al. from 43.6 to 87.5 at the mean follow-up of 24 months [19]. With the improvement of the mean Lysholm score from 54 to 86 at a mean follow-up of 39 months reported in this study, the results are comparable with the abovementioned literature, with longer follow-up. On contrary, in the study of Xinxian et al., the authors reported improvement of the Lysholm score in arthroscopically treated group from the preoperative mean of 76.6 to 86.3 at the mean of 33.3 months follow-up [20]. While authors stated that all patients “complained of symptomatic knee joint pain and/or joint limitation,” a lot higher preoperative Lysholm score than in the rest of the literature can be seen, making direct comparison of outcomes difficult [7, 19, 20]. The authors failed to find any studies assessing the functional outcomes of arthroscopic popliteal cyst treatment by the means of VAS perceived satisfaction score, and therefore, a comparison of this functional outcome with the literature was not possible.

 Regarding the distribution of concomitant lesions, the presented reported case series does not differ significantly from previous reports. Sansone et al. has found intra-articular lesions in 94% of popliteal cyst cases [1]. Specifically, the most prevalent were medial meniscus tears, chondral lesions, and anterior cruciate ligament tears, similar to this study cohort. Likewise, Johnson et al. has reported an incidence of 71% for meniscal tears and of 81% for chondral degenerative changes with cases of communicating popliteal cysts [2]. Yang et al. has reported that abandoning of intra-articular lesion management resulted in a 7.5-fold increase in the cyst’s recurrence rate [7]. This observation stays in agreement with the findings of Rupp et al. [21]. In their study, ten of 11 patients with recurrent cyst had grade III–IV chondral lesions, whereas none of five patients with cyst resolution demonstrated such a Outerbridge Classification grade [21]. Analogously, the risk recurrence of the cyst in the current study was significantly higher in cases with III–IV grade chondral lesions. This finding supports the importance of the treatment of concomitant lesions in the pathogenesis of popliteal cysts. The proposed explanation of this phenomenon is that in case of irreparable intra-articular lesions, persistent excessive synovial fluid production increases the recurrence risk [1, 5].

**Fig. 4:**
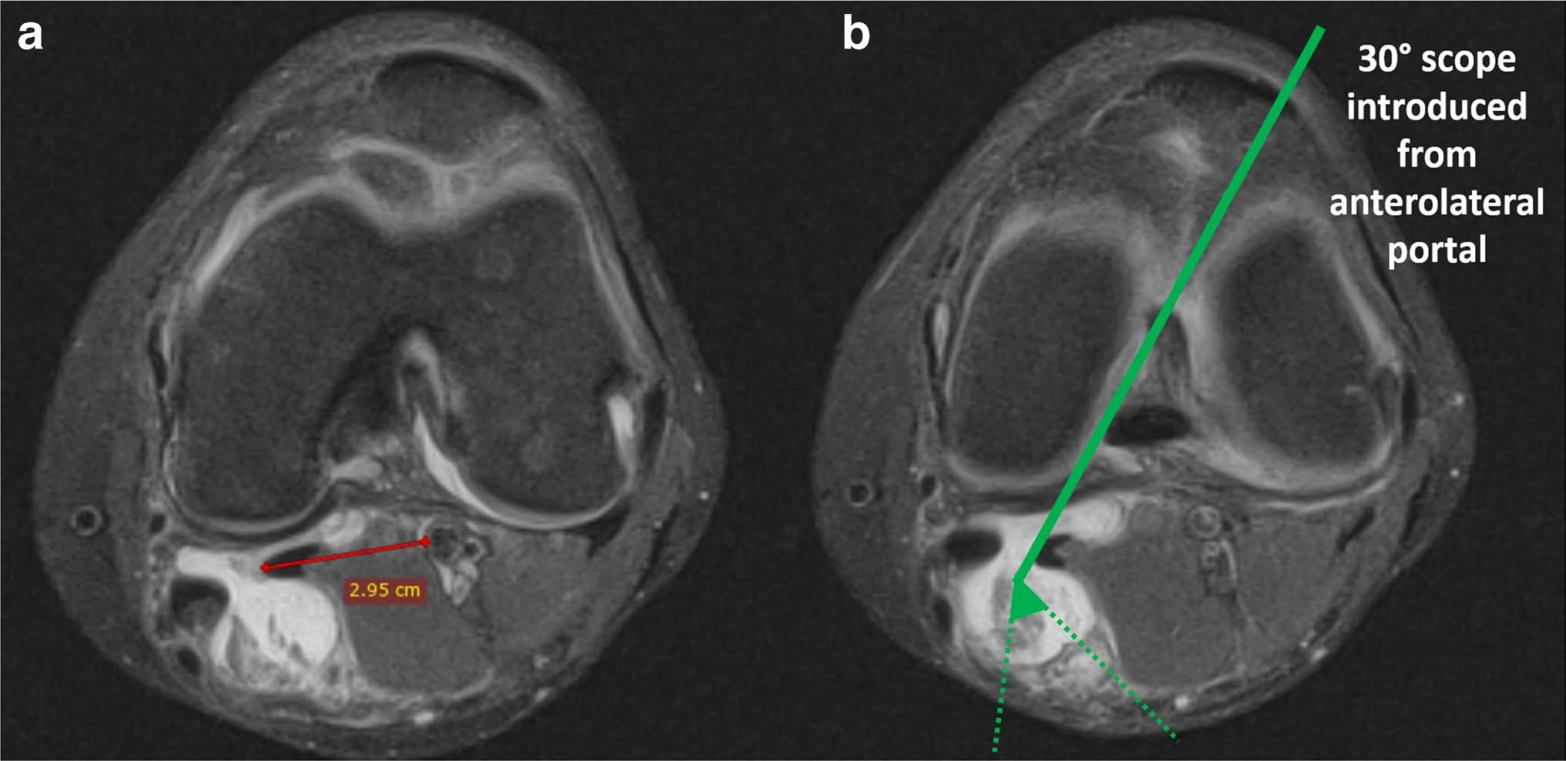
Transverse MRI scans of the left knee concerning spatial relationship between the popliteal cyst and neurovascular bundle: (**a**) in case of typical popliteal cyst localization in the gastrocnemius–semimembranosus bursa, the neurovascular bundle is protected by the medial head of gastrocnemius. (**b**) As can be seen, if the surgeon is working only within tissues possible to visualize from anterolateral portal after trans-notch maneuver, the neurovascular bundle is out of the surgical field

Concerns of the safety of the procedure described in the current manuscript may occur due to relative proximity of neurovascular bundle. However, in case of the typical popliteal cyst localization in the gastrocnemius–semimembranosus bursa, the neurovascular bundle is protected by the medial head of gastrocnemius (Fig. 4). In accordance with this protection, confirmed by the means of ultrasonography and MRI preoperatively, no persistent complications were observed in any of analyzed patients, the same as in the studies of Gu et al., Lie et al., Ahn et al., and Chen et al. [14–17]. What is more, if the surgeon is using a 30° arthroscope and is working only within tissues possible to visualize from the anterolateral portal after a trans-notch maneuver, the neurovascular bundle is out of the surgical field (Fig. 4). It is also important to stay within fibrous tissues and to not excise muscular tissue separating the working area from the neurovascular bundle.

### Limitations

The first limitation of this study was the wide dispersion of the follow-up, ranging from 12 to 71 months. Also, a relatively high percentage of patients (17.8%) were lost to follow-up. What is more, the age group in this study is younger than in the literature [14–17]. This may be due to the fact, that the senior author’s practice is performed in the sports clinic with more younger patients with overuse syndromes or sports injuries. However, as the study group were consecutive patients, we believe that it accurately represented everyday clinical practice. Nevertheless, the results of this study should not be extrapolated outside the assessed age group. This study possesses some strengths to be highlighted as well. First, the current study reports on one of the largest cohorts currently available in the literature of popliteal cysts treated with arthroscopic technique on the results of the procedure and intraoperative assessment of the cyst wall and valve morphology and concomitant intra-articular pathologies [5, 22]. Second, this series is both complete and consecutive, avoiding bias caused by inclusion of selected patients with better prognosis (sampling bias) [23]. Moreover, the follow-up protocol was strict, including thorough functional and restrictive ultrasonographic assessment criteria for recurrence (any fluid in popliteal space).

## Conclusions

Our findings demonstrate arthroscopic popliteal cyst treatment had a low recurrence rate and good functional outcomes, and further severe chondral lesions (Outerbridge grade III vs. IV) lead to a significant increased risk of cyst recurrence.

## Data Availability

All available data can be send by the corresponding author on request.

## Code availability

This is not applicable.
